# Synthesis and Physicochemical Characterization of Sodium-Based Electrolytes: A Preliminary Study

**DOI:** 10.3390/ma19102127

**Published:** 2026-05-19

**Authors:** André Pinto, Caroline Maria Bezerra de Araujo, Maria Manuela Silva, Mariana Fernandes

**Affiliations:** 1 Chemistry Research Centre-Vila Real (CQ-VR), University of Trás-os-Montes e Alto Douro, 5000-801 Vila Real, Portugal; 2 Centre of Chemistry, University of Minho, 4710-057 Braga, Portugal; carolinemariaba@gmail.com (C.M.B.d.A.); nini@quimica.uminho.pt (M.M.S.)

**Keywords:** solid electrolytes, eco-friendly ormolytes, sodium-ion electrolytes, sustainable electrochemical devices

## Abstract

Sodium-ion-based polymer electrolytes have emerged as an essential technology for the next generation of solid-state batteries, offering the possibility of greater safety and mechanical flexibility. This work aimed to prepare eco-friendly ormolytes based on a biohybrid host matrix, which were doped, for the first time, with a wide range of NaTFSI concentrations. The matrix consists of short poly(ε-caprolactone) segments covalently bonded to siliceous domains via urethane linkages. The samples obtained were thin and transparent films. They were characterized by means of thermogravimetric analysis (TGA) and X-ray diffraction (XRD), and the films exhibited an amorphous character over the entire composition range. Ionic conductivity measurements were performed, and at room temperature for n = 10, the ionic conductivity was 2.44 × 10^−3^ mS.cm^−1^. The highest ionic conductivity value of 1.78 × 10^−2^ mS.cm^−1^ (n = 10) was obtained at 62.0 °C. To access the cation/urethane interactions, Fourier transform infrared (FT-IR) spectroscopy was employed, and it was noted that the global profile was slightly altered with the incorporation of salt, in which more interactions were observed for the more concentrated samples. Thus, the proposed material may be promising in the development of more sustainable and environmentally friendly electrochemical devices with Na ions.

## 1. Introduction

The importance of batteries in everyday life [1] is well known. Batteries play a pivotal role in energy storage applications, and their widespread use across diverse sectors underscores their importance in enabling the functionality, mobility, and resilience of modern technological systems.

Although lithium-ion batteries (LIBs) have been successfully commercialized, the limited and geographically concentrated natural abundance of lithium raises sustainability concerns. In contrast, sodium-ion batteries (NIBs) offer cost and safety advantages, because sodium—an abundant and widely distributed alkali metal—can serve as the charge carrier in a system that is largely a drop-in alternative to LIBs due to their similar materials and manufacturing methodologies [2,3,4,5,6]. NIBs were first studied alongside LIBs in the 1970s and 1980s but were consequently abandoned because of the rapid commercial success of LIBs.

NIBs represent one of the most mature post-lithium technologies, delivering strong environmental and electrochemical performance while minimizing the use of scarce resources. Sodium can be readily extracted from seawater, and its processing can leverage much of the existing lithium-ion manufacturing infrastructure, facilitating NIBs’ potential industrial scalability [2,4,7,8].

One important issue regards the type of elecctrolytes [9], with polymer electrolytes (PEs) being a key type. PEs have emerged as an essential technology for the next generation of NIBs, offering a pathway to enhanced safety and mechanical flexibility. Unlike conventional liquid electrolytes, which are flammable and prone to leakage, solid polymer electrolytes (SPEs) [10,11] provide a non-volatile and safer alternative. The most common electrolyte formulations for NIBs are based on sodium salts, such as sodium perchlorate (NaClO_4_) or sodium hexafluorophosphate (NaPF_6_), in carbonate ester solvents, particularly propylene carbonate (PC) [12]. Sodium bis(trifluoromethanesulfonate) imide (NaTFSI) complexed with poly(ethylene oxide) (POE) was successfully processed into membranes using a solvent-free hot-pressing technique, with the EO:Na molar ratio varied. These membranes exhibit ionic conductivities on the order of 10^−3^ S cm^−1^ at temperatures above 70 °C [13]. The ionic conduction in SPEs occurs primarily in the amorphous phase of the polymer through segmental motion, facilitating the transport of sodium ions. While they offer the advantage of suppressing sodium dendrite growth and enabling simpler cell design, challenges remain in achieving sufficient ionic conductivity at room temperature and ensuring long-term interfacial stability with electrodes. Current research focuses on developing advanced polymer blends, copolymers, and composite systems to overcome these limitations, positioning polymer electrolytes as a promising solution for safe and durable sodium-based energy storage.

In the past few years, the sol-gel process [14] has been widely adopted for the synthesis of ion-conducting organic–inorganic hybrid composites, known as ormolytes (organically modified silicate electrolytes) [15]. These materials are particularly promising for application in solid-state electrochemical devices, including sensors, systems for energy and data storage, batteries, and electrochromic and photovoltaic devices [16]. Their attractiveness stems from a unique combination of properties: they exhibit excellent mechanical, chemical, and thermal stability while also being capable of accommodating high concentrations of ionic salts. This structural versatility allows for the fine-tuning of their ionic conductivity and electrochemical performance, making them highly suitable for advanced energy technologies. Several examples can be found in the literature: a hybrid structure is composed of a siliceous framework to which poly(oxyethylene) (POE) chains with variable chain length are covalently bonded through urea linkages and doped with different amounts of sodium triflate [17]; POE chains CH_3_-terminated with approximately 17 oxyethylene repeat units and covalently bonded to the siliceous network by means of a single urethane group [18]; or di-urethane cross-linked poly(ε-caprolactone) (PCL(530))/siloxane matrix doped for the first time with different amounts of sodium triflate (NaCF_3_SO_3_.xH_2_O) [19]. Poly(ε-caprolactone) (PCL) is a nontoxic and biodegradable polymer widely used in biomedical applications. Its potential as a host for ionic conduction arises from the Lewis basicity of its ester oxygen groups, which can coordinate cations.

This study aimed to produce a new series of eco-friendly ormolytes based on a biohybrid host matrix, which were doped, for the first time, with a wide range of NaTFSI concentrations.

Over the past decade, organic–inorganic hybrid electrolytes (ormolytes) based on poly(ε-caprolactone) (PCL) and siloxane networks synthesized via sol-gel routes have emerged as promising candidates for solid-state electrochemical applications. PCL–siloxane hybrids doped with lithium or sodium salts, including LiTFSI and NaCF_3_SO_3_, are known to provide mechanically robust and thermally stable matrices for ion transport. However, their practical implementation is hindered by intrinsically low ionic conductivity and insufficient ion dissociation—limitations that are particularly pronounced in sodium-based systems due to strong ion pairing and weak polymer–salt interactions. Moreover, sodium-conducting ormolytes remain significantly underdeveloped compared to their lithium-based counterparts despite the urgent demand for sustainable and cost-effective sodium-based energy storage technologies.

Herein, we report a new class of PCL–siloxane ormolytes incorporating sodium bis(trifluoromethanesulfonyl)imide (NaTFSI), specifically engineered to promote efficient sodium-ion transport through enhanced salt dissociation and improved compatibility with the polymer network. The incorporation of the highly delocalized TFSI^−^ anion effectively suppresses the ion pairing relative to conventional salts such as NaCF_3_SO_3_, resulting in markedly improved ionic conductivity.

The matrix consists of short poly(ε-caprolactone) segments (PCL(530), where 530 is the average molecular weight in g·mol^−1^) covalently bonded to siliceous domains via urethane (NHC(O)O) linkages. The resulting materials are denoted as d-PCL(530)/siloxane*n*’NaTFSI, where *d* stands for “di” and *n*’ represents the number of ester repeat units (C(O)(CH_2_)_5_O) of PCL(530) per sodium ion. The samples, obtained as thin, transparent films, were examined by means of thermogravimetric analysis (TGA), X-ray diffraction (XRD), and atomic force microscopy (AFM). The wettability and electrochemical impedance spectroscopy (EIS) characteristics were also evaluated. To access the cation/urethane interactions, Fourier transform infrared (FT-IR) spectroscopy was employed.

## 2. Materials and Methods

### 2.1. Materials and Synthesis

α,ω-hydroxyl poly(ε-caprolactone) (PCL(530), Fluka (St. Louis, MO, USA), average molecular weight 530 g mol^−1^), 3-isocyanatepropyltriethoxysilane (ICPTES, Fluka, St. Louis, MO, USA) and sodium bis(trifluoromethylsulfonyl)imide (NaTFSI) were used as received. Ethanol (CH_3_CH_2_OH, Merck, Darmstadt, Germany) and tetrahydrofuran (THF, Merck) were stored over molecular sieves. Mili-Q water was used in all experiments.

The synthesis was performed in two steps according to the method described elsewhere [20]. The most relevant experimental details can be found in Table 1.

### 2.2. Thermogravimetric Analysis

TGA measurements were performed using an STA 449 F3 Jupiter instrument (NETZSCH-Gerätebau GmbH, Selb, Germany) equipped with Proteus software (version 7.1). Measurements were carried out over a temperature range of 25–700 °C at a heating rate of 10 °C min^−1^. Xerogel samples with masses between 5 and 10 mg were placed in alumina crucibles. Nitrogen was used both as the purge gas (50 mL min^−1^) and as the protective gas (20 mL min^−1^).

### 2.3. X-Ray Diffraction

XRD measurements were performed at room temperature using a Philips X’Pert MPD powder diffractometer (Malvern PANalytical, Almelo, The Netherlands) with monochromated CuKα radiation (λ = 1.541 Å). Data were collected over a 2θ range of 10–50°. The samples were analyzed without any prior thermal treatment.

### 2.4. Fourier Transform Infrared Spectroscopy

FT-IR spectra were recorded at room temperature using an IRAffinity-1S Fourier transform infrared spectrometer with LabSolutions IR software (Version 1.50, Shimadzu, Kyoto, Japan). Spectra were collected in the range 4000–400 cm^−1^ by averaging at least 128 scans at a resolution of 2 cm^−1^. Xerogel samples were used for infrared analysis. To analyze complex band envelopes and identify the underlying component bands, an iterative least-squares curve-fitting procedure was applied using PeakFit software (version 4).

### 2.5. Contact Angle Measurements

Contact angle measurements were performed using a Krüss DSA25S Drop Shape Analyzer (Hamburg, Germany) in a temperature-controlled chamber at 25 ± 1 °C, employing the sessile drop method. Wettability was evaluated using 5 µL droplets of ultra-pure distilled water. Contact angles were determined from digital images captured by a video camera using Young–Laplace fitting, and data analysis was carried out with ADVANCE software (Version 1.15.0.35501). Ten measurements were performed for each sample.

### 2.6. Atomic Force Microscopy

AFM measurements were carried out in tapping mode using a Nano-Observer AFM microscope (CSInstruments AFM Microscopes, Les Ulis, France), operating at a resonance frequency of 60 kHz and a spring constant of 0.3 N m^−1^. A super-sharp Si HQ:NSC19/FORTA probe (NanoAndMore USA Corp., Woburn, MA, USA) was employed. Image processing, including flattening, line-noise removal, and low-pass filtering, was performed using Gwyddion software (version 2.52) to improve image quality.

### 2.7. Ionic Conductivity

The ionic conductivity of each ormolyte was measured via electrochemical impedance spectroscopy (EIS) using an Autolab PGSTAT-12 potentiostat/galvanostat (Eco Chemie, Utrecht, The Netherlands). A symmetrical cell was assembled using ion-blocking gold electrodes (10 mm diameter, >99.95% purity; Goodfellow, Delson, QC, Canada), with a small amount of ormolyte sandwiched between the electrodes. The cell was placed in a Büchi TO51 tube oven (Flawil, Switzerland), and a type K thermocouple was positioned near the ormolyte disk to monitor the sample temperature. Bulk ionic conductivity was determined by complex impedance spectroscopy during heating cycles from room temperature to approximately 100 °C over a frequency range of 65 kHz to 500 mHz.

The ionic conductivity (*σ_i_*) was quantified using Equation (1):

(1) $$\sigma_{i}=\frac{d}{R_{b}\times A}$$

Here, *R_b_* represents the bulk resistance of the sample, *d* represents its thickness, and *A* stands for the area.

## 3. Results and Discussion

From the syntheses carried out, transparent and flexible films were obtained, which were characterized by the techniques described in Section 2.

### 3.1. Thermal and Structural Analysis of the d-PCL(530)/siloxane_n_NaTFSI Ormolytes

**Figure 1:**
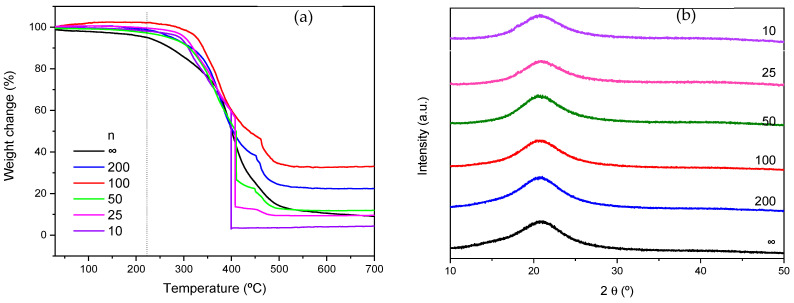
Thermograms (**a**) and diffractograms (**b**) of the films obtained.

Figure 1 presents the thermograms (a) and diffractograms (b) of the d-PCL(530)/siloxane_n_NaTFSI ormolytes. All the samples analyzed are thermally stable up to 225 °C (the dashed line in Figure 1a is only a guide for the eye). Above this temperature (marked with a dashed line in the Figure 1a), decomposition begins, particularly for samples containing higher amounts of the guest salt. The films exhibit an amorphous character over the entire composition range studied. The diffractograms (Figure 1b) show a broad band centered at 21°, which is attributed to diffraction from the siliceous domains [21].

FTIR spectroscopy is a powerful technique for investigating the interactions between sodium salts and the host d-PCL(530)/siloxane hybrid. This matrix provides three types of donor atoms: (i) carbonyl oxygen atoms from urethane cross-links, (ii) ester carbonyl oxygen atoms from the PCL(530) chains, and (iii) ether oxygen atoms from the central oxyethylene segment of PCL(530).

Upon incorporation into the d-PCL(530)/siloxane framework, Na^+^ species may either interact with “free” urethane/ester C=O groups or coordinate with hydrogen-bonded carbonyl groups. Coordination of the cation to urethane and ester carbonyl groups is reflected in the amide I region, which corresponds to the carbonyl stretching vibration commonly observed in polyamides. Due to its sensitivity to hydrogen bonding, the amide I band typically consists of multiple components associated with different carbonyl environments (aggregates). Since the absorption coefficients of these components may differ, only variations within the same component as a function of salt concentration should be compared [22,23].

**Figure 2:**
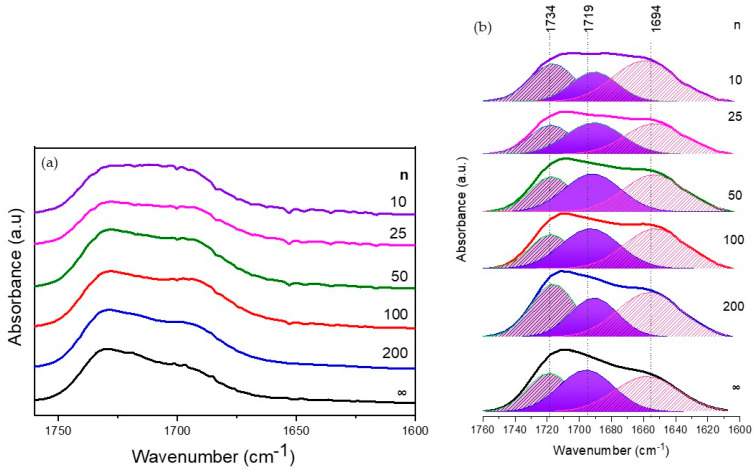
ATR spectra in the amide I region of the d-PCL(530)/siloxane_n_NaTFSI ormolytes (**a**). Curve fitting of the “amide I” region of d-PCL(530)/siloxane-based samples doped with NaTFSI (**b**).

The amide I spectra of the films are presented in Figure 2a, and their deconvolution is shown in Figure 2b. The overall spectral profile changes slightly with increasing salt content, becoming broader for the most concentrated sample (n = 10). Notably, the band corresponding to “free” urethane C=O groups (around 1760 cm^−1^) is absent.

The band at 1734 cm^−1^ is assigned to ester C=O stretching vibrations in amorphous PCL(530) chains. Its relative contribution increases significantly upon initial doping, from approximately 27.1% in the undoped sample to 33.6% for n = 200. At intermediate concentrations (n = 100, 50, and 25), this contribution decreases and stabilizes at similar values (23.2%, 25.8%, and 25.9%, respectively) before increasing again for the most concentrated sample (30.3% at n = 10).

The absorption at 1719 cm^−1^ is attributed to hydrogen-bonded oxyethylene/urethane aggregates. Interestingly, its trend is opposite to that observed at 1734 cm^−1^. From the undoped sample to n = 200, the contribution decreases significantly (from 36.8% to 26.9%). For intermediate concentrations (n = 100, 50, and 25), it remains relatively constant (37.5%, 36.2%, and 36.8%), followed by a marked decrease at n = 10 (24.6%).

The band at 1694 cm^−1^ is associated with C=O stretching in more ordered hydrogen-bonded aggregates, including ester/urethane and urethane/urethane interactions. The relative contribution of this band increases upon salt addition, with a more pronounced effect at the highest concentration (n = 10).

Overall, these changes contribute to a modification of the amide I profile, indicating that the strongest interactions occur at higher salt concentrations. The FTIR results indicate that Na^+^ ions preferentially interact with carbonyl groups of the host matrix, particularly urethane and ester functionalities, leading to significant rearrangements in hydrogen-bonding networks. Initial salt incorporation disrupts hydrogen-bonded aggregates, as evidenced by the decrease in the 1719 cm^−1^ band, while higher salt concentrations promote the formation of more ordered aggregates (1694 cm^−1^ band). The broadening of the amide I band and the increase in ordered aggregate contributions at n = 10 indicate stronger ion–polymer interactions and possible structural reorganization of the hybrid network. The disappearance of the “free” urethane C=O band suggests that most available carbonyl groups participate in coordination or hydrogen bonding upon salt addition.

### 3.2. Wettability of the d-PCL(530)/siloxane_n_NaTFSI Ormolytes

**Figure 3:**
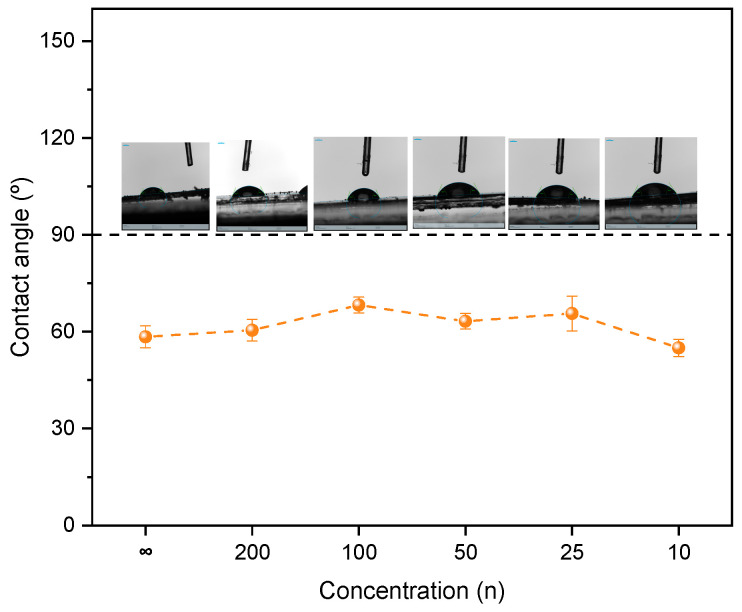
Contact angle values obtained for the d-PCL(530)/siloxane_n_NaTFSI ormolytes.

To evaluate the wettability behavior of the NaTFSI-doped d-PCL(530)/siloxane system, the static contact angle was measured. The samples exhibit a hydrophilic character, with contact angle values ranging from 55° (for n = 10) to 68° (for n = 100). No significant change is observed upon salt addition, since the undoped sample shows a contact angle of 58° (Figure 3). As seen, the inset images in the Figure 3 are representative of the water drop obtained for each sample. These values are in accordance with the values obtained for di-ureasils, d-U′(400), which include organic chains with approximately 6.1 CH(CH_3_)CH_2_ repeat units and present a contact angle of 66.29 ± 5.62° [24], and for two di-ureasils incorporating oxyethylene segments with average molecular weights Y = 600 and 900 g mol^−1^, where the values obtained were 50.64 ± 24.08° and 61.36 ± 13.30°, respectively [23].

### 3.3. Morphology of the d-PCL(530)/Siloxane_n_NaTFSI Ormolytes

**Figure 4:**
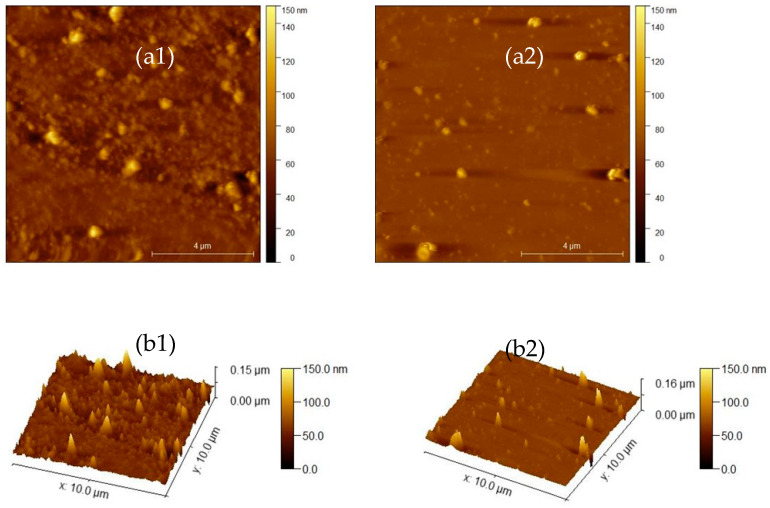
2D (**a1**,**a2**) and 3D (**b1**,**b2**) AFM images of d-PCL(530)/siloxane_n_NaTFSI ormolytes for n = 200 (**a1**,**b1**) and n = 10 (**a2**,**b2**).

AFM is a high-resolution imaging technique that maps surface structures at the nanoscale by scanning a sharp probe across a sample. Representative images for n = 200 and n = 10 are presented in Figure 4. The 2D images reveal a uniform and homogeneous surface, indicating consistent morphology across the analyzed area. From the 3D images it is possible to calculate the roughness of the sample. The roughness values are similar for the samples with n = 200, 100, and 25 (4.22, 4.38, and 4.72 nm, respectively), while slightly lower values are observed for the samples with n = 10 (2.68 nm) and n = 50 (1.95 nm), suggesting a more uniform film growth in the last examples.

Apparently, the incorporation of the salt does not affect the Ra value, being in fact much lower than that reported for di-ureasils systems doped with a mixture of lithium salts and commercial ionic liquid [25].

### 3.4. Ionic Conductivity of the d-PCL(530)/siloxane_n_NaTFSI Ormolytes

**Figure 5:**
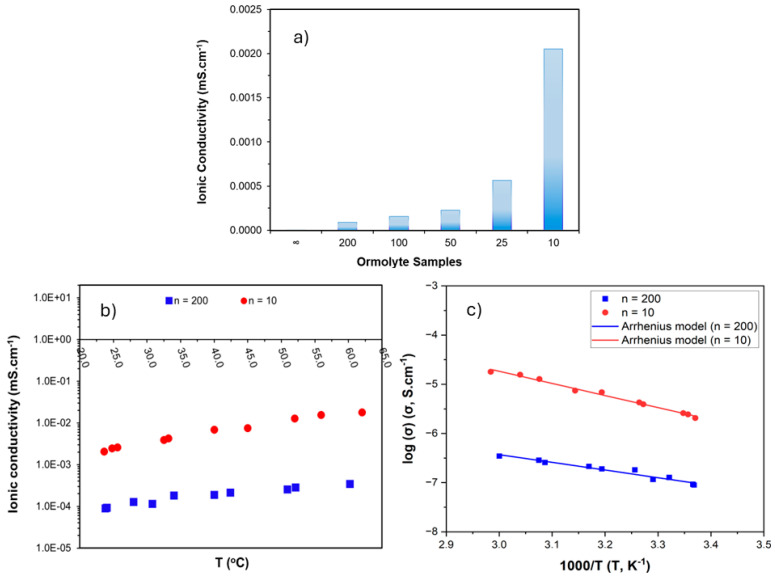
Ionic conductivity values of the ormolytes at room temperature (**a**); ionic conductivity (samples n = 200 and 10) at different temperatures (**b**); temperature-dependent manner via the Arrhenius model for samples n = 200 and n = 10 (**c**).

EIS analyses of the ormolytes produced were conducted at room temperature, and the results in terms of ionic conductivity are exhibited in Figure 5a. From the graph, it is noted that as the salt concentration increased in the ormolytes, the ionic conductivity values gradually increased. High ionic conductivity is one of the most important properties that a polymer electrolyte should have. The higher the amount of sodium salt in the ormolytes, the greater the ionic conductivity values. At room temperature (~24 °C), the values of ionic conductivity ranged between 9.27 × 10^−5^ mS.cm^−1^ (n = 200) and 2.44 × 10^−3^ mS.cm^−1^ (n = 10). When compared with previously reported systems, these values are significantly higher. For instance, the d-PCL(530)/siloxane NaCF_3_SO_3_·1.4H_2_O biohybrids reported by M. Fernandes et al. [19] exhibited a maximum ionic conductivity of only 4.1 × 10^−7^ mS·cm^−1^ (at 30 °C, n = 6). Similarly, the most conductive ormolyte U(600)_10_NaCF_3_SO_3_ described by S. M. Gomes Correia et al. [17] showed a conductivity of 3.6 × 10^−7^ mS·cm^−1^ at room temperature. Overall, the ionic conductivities achieved in the present work are two to four orders of magnitude higher than those reported for comparable sodium-based ormolyte and biohybrid systems. This substantial improvement highlights the enhanced ion transport properties of the developed materials.

As seen in the figure, it is clear that the ionic conductivity values increased continuously with increasing temperature until they reached values close to 3.46 × 10^−4^ mS.cm^−1^ (n = 200) at 60.2 °C and 1.78 × 10^−2^ mS.cm^−1^ (n = 10) at 62.0 °C. The increase in ionic conductivity as a function of increasing temperature is possibly due to increased thermal movement of the polymer chain segments, enlarged free volume with increasing temperature, and decreased viscosity of the polymer matrix [26,27].

The ionic conductivity values observed in the present work were significant when compared to other solid sodium electrolytes reported in the literature. For instance, Youcef et al. [28] prepared a polymer electrolyte with PEO/functionalized ethyl cellulose and sodium fluorosulfonylimide (NaFSI). The electrolyte exhibited high electrochemical stability and an ionic conductivity value of around 10^−4^ mS·cm^−1^ at room temperature.

The trend observed in Figure 5c for the ormolyte samples n = 200 and n = 10 in the linearized data with increasing temperature is consistent with the Arrhenius equation, presented by Equation (2).

(2) $$\sigma =\sigma_{0}\;exp\left(\frac{-E_{a}}{k_{B}T}\right)$$

where *E_a_* represents the activation energy (eV), *k_B_* is given by the Boltzmann constant (8.617 × 10^−5^ eV.K^−1^), *T* is the temperature (K), and *σ*_0_ is the pre-exponential factor [29]. Table 2 presents the activation energy values calculated from the Arrhenius equation, as well as the respective statistical parameters obtained.

Overall, it is observed that the increase in the NaTFSI concentration in the ormolyte leads to a higher number of charge carriers, which contributes to the increase in ionic conductivity values. Nevertheless, there is evidence that an increase in salt concentration simultaneously promotes stronger ion–polymer interactions as well, along with possible formation of ion pairing, causing ion mobility to be more restricted at higher salt contents. This results in an increase in the activation energy value, reflecting the difficulty of ion transport within the matrix [13].

## 4. Conclusions

In the present work, a set of transparent and flexible films were prepared using a biodegradable polymer cross-linked through a urethane linkage to a siliceous domain via the sol-gel method. The samples were doped with different amounts of guest sodium salt, NaTFSI. The films obtained were amorphous, thermally stable as far as expected, homogeneous and hydrophilic. The FTIR results demonstrate that Na^+^ incorporation induces significant modifications in the carbonyl environment of the d-PCL(530)/siloxane hybrid. Variations in the amide I band components indicate a concentration-dependent interplay between disruption and reformation of hydrogen-bonded aggregates. The decrease in hydrogen-bonded oxyethylene/urethane structures and the concurrent increase in more ordered aggregates at high salt content suggest progressive structural reorganization. Overall, the data support strong coordination between Na^+^ ions and carbonyl groups, particularly at higher salt concentrations.

Ionic conductivity is a critical factor to be considered when assessing the potential of an SPE for battery applications. With increasing temperature due to increased charge mobility, this conductivity also depends on the sodium salt content. When the salt content increases, the ionic conduction behavior improves. An improvement in ionic conductivity is observed with increasing temperature and sodium salt content due to increased mobility of the mobile charge carriers and the polymer chain.

Overall, this work aimed to prepare and characterize a potential candidate for SPE, providing information on the mechanisms of ionic transport in biohybrid ormolytes. Further validation with an electrochemical device, which is very important, will be addressed in future work.

## Figures and Tables

**Table 1:** Relevant experimental details of the synthetic procedure employed.

Mass PCL (530) (g)	Volume ICPTES (μL)	Mass NaTFSI (g)	n	Volume Ethanol (μL)	Volume H_2_O (μL)
1.0150	948	-	∞	945	104
1.0034	938	0.0105	200	895	102
1.0055	940	0.0213	100	896	103
1.0020	936	0.0424	50	893	102
1.0032	937	0.0849	25	894	102
1.0330	965	0.2186	10	921	105

**Table 2:** Parameters calculated from the Arrhenius equation.

Parameters	Ormolyte n = 200	Ormolyte n = 10
Slope	−1.560	−2.459
*E_a_* (eV)	0.310	0.488
Residual Sum of Squares	0.0171	0.0102
Adjusted R^2^	0.950	0.989

## Data Availability

The original contributions presented in this study are included in the article. Further inquiries can be directed to the corresponding author.
